# A Lumenal Loop Associated with Catalytic Asymmetry in Plant Vacuolar H^+^-Translocating Pyrophosphatase

**DOI:** 10.3390/ijms222312902

**Published:** 2021-11-29

**Authors:** Viktor A. Anashkin, Alexander A. Baykov

**Affiliations:** Belozersky Institute of Physico-Chemical Biology, Lomonosov Moscow State University, 119899 Moscow, Russia; victor_anashkin@belozersky.msu.ru

**Keywords:** membrane pyrophosphatase, H^+^ pump, energy coupling, molecular dynamics, kinetic co-operativity, *Vigna radiata*, plant vacuole

## Abstract

Membrane-integral inorganic pyrophosphatases (mPPases) couple pyrophosphate hydrolysis with H^+^ and Na^+^ pumping in plants and microbes. mPPases are homodimeric transporters with two catalytic sites facing the cytoplasm and demonstrating highly different substrate-binding affinities and activities. The structural aspects of the functional asymmetry are still poorly understood because the structure of the physiologically relevant dimer form with only one active site occupied by the substrate is unknown. We addressed this issue by molecular dynamics (MD) simulations of the H^+^-transporting mPPase of *Vigna radiata*, starting from its crystal structure containing a close substrate analog (imidodiphosphate, IDP) in both active sites. The MD simulations revealed pre-existing subunit asymmetry, which increased upon IDP binding to one subunit and persisted in the fully occupied dimer. The most significant asymmetrical change caused by IDP binding is a ‘rigid body’-like displacement of the lumenal loop connecting α-helices 2 and 3 in the partner subunit and opening its exit channel for water. This highly conserved 14–19-residue loop is found only in plant vacuolar mPPases and may have a regulatory function, such as pH sensing in the vacuole. Our data define the structural link between the loop and active sites and are consistent with the published structural and functional data.

## 1. Introduction

Membrane-bound inorganic pyrophosphatases (mPPase; EC 7.1.3.1, formerly 3.6.1.1), a relict transporter found in plants and microbes, uses pyrophosphate (PP_i_) energy to translocate H^+^ and Na^+^ across membranes [1,2,3,4,5,6]. The mPPase superfamily is divided into K^+^-independent and K^+^-dependent families [4]. The former family includes only H^+^ transporting mPPases, whereas the K^+^-dependent family encompasses both H^+^ and Na^+^ transporters. Plants posess only H^+^-transporting mPPases of both families, and their overexpression is a well-documented approach to enhancing plant tolerance to abiotic stress [7]. 

The mPPase is one of the simplest cation transporters and consists of one 66–89-kDa polypeptide that folds into 15–17 membrane-spanning α-helices. The enzyme is homodimeric, with two catalytic sites separated in space and facing the cytoplasm. According to the crystal structures [8,9], the nucleophilic water molecule that attacks PP_i_ and releases a proton as a by-product is located just at the entrance to the ion conductance channel, suggesting a unique “direct coupling” mechanism of H^+^ pumping [10]. All other non-redox H^+^ pumps use “indirect coupling” mechanisms, wherein the transported H^+^ ion is not directly involved in the chemical reaction but comes from the solution [10]. In such transporters, including various ATPases, the reaction site and the conductance channel are separated in space, and the energy is input via a conformational strain. A mechanism with indirect coupling was also suggested for mPPase [11] but appeared to be inconsistent with the kinetics and magnitude of charge transfer across the membrane during PP_i_ hydrolysis [12]. 

While mechanistically different from the ATPases, mPPase may nevertheless use elements of the indirect coupling in its mechanism. Kinetic studies of various mPPases from the K^+^-dependent and K^+^-independent families indicated that the second active site is saturated at much larger substrate concentrations, resulting in a diminished PP_i_ hydrolysis rate than with the monosubstrate enzyme [13,14,15,16]. The same trend was observed in the H^+^ transport reaction [13,16]. PP_i_ analogs containing N or C instead of O in the bridge position also bind asymmetrically and similarly modulate substrate binding and catalysis in the partner subunit [15]. Moreover, the novel inhibitor of mPPase N-[(2-amino-6-benzothiazolyl)methyl]-1H-indole-2-carboxamide (ATC), which bound at the channel exit (i.e., far from the active site) of one subunit, completely inactivated both subunits [17]. The substrate-binding asymmetry is modulated by the cofactor Mg^2+^ [15]. 

The structural aspects of subunit cooperation in mPPase are still poorly understood. It was speculated that the subunits undergo turnover-linked cyclical oscillations between two conformations [14] to store and consume the conformational energy. This seminal idea requires, however, a structural verification. Several crystal structures of two K^+^-dependent mPPases in various states have been solved [18]. One of them is the plant vacuolar H^+^-transporting mPPase of *Vigna radiata* (Vr-mPPase), the other is the Na^+^-transporting mPPase of the thermophilic bacterium *Thermotoga maritima* (Tm-mPPase). The active-site bound ligands for both mPPases included the PP_i_ analog imidodiphosphate (IDP), containing NH instead of O in the bridge position, and two or one P_i_ or WO_4_ molecule(s) per active site. Most structures also contained two to five Mg^2+^ ions and one K^+^ ion per active site. These structures were historically a breakthrough in mPPase studies, but they contain phosphate ligands in both active sites and therefore are ‘potentially not mechanistically relevant at typical PP_i_ concentrations’ [18]. It is thus crucial for understanding the functional asymmetry to determine the physiologically relevant structure with the substrate in only one active site, which may not be a simple task for protein crystallography.

In the present study, we predicted, using molecular dynamics (MD) simulations, the solution structures of Vr-mPPase containing IDP in only one active site per dimer and in the IDP-free form. The starting point of our simulations was the crystal structure of this enzyme containing IDP in both active sites [8]. The predicted structures support and further extend the concept of active site mPPase cooperation in the substrate-binding step and complement the known structural data.

## 2. Results

### 2.1. Molecular Dynamics Simulations of mPPase Dimer

A crystal structure of IDP-complexed Vr-mPPase (2.35 Å resolution) [8] was selected for MD simulations because of its higher resolution in comparison with the Tm-mPPase structure (3.5 Å resolution) [9] and simpler transport specificity (only H^+^ ion). First, mPPase structure containing IDP in each subunit was simulated in a virtual membrane, mimicking plant vacuolar membrane, to relieve structural distortions of the crystalline state (Figure S1). The simulations were performed for 200 ns, with snapshots saved every 10 ps, and nearly complete equilibration was achieved after 80–100 ns (Figure S2). The simulated Vr-mPPase structure demonstrated small but significant variations from the crystal structure. Next, the IDP molecule with the associated K^+^ and three Mg^2+^ ions were removed from one or both subunits in the crystal structure, and the simulation was repeated. Interestingly, the removed K^+^ ion re-occupied its initial position in all structures during the simulation procedure.

**Figure 1:**
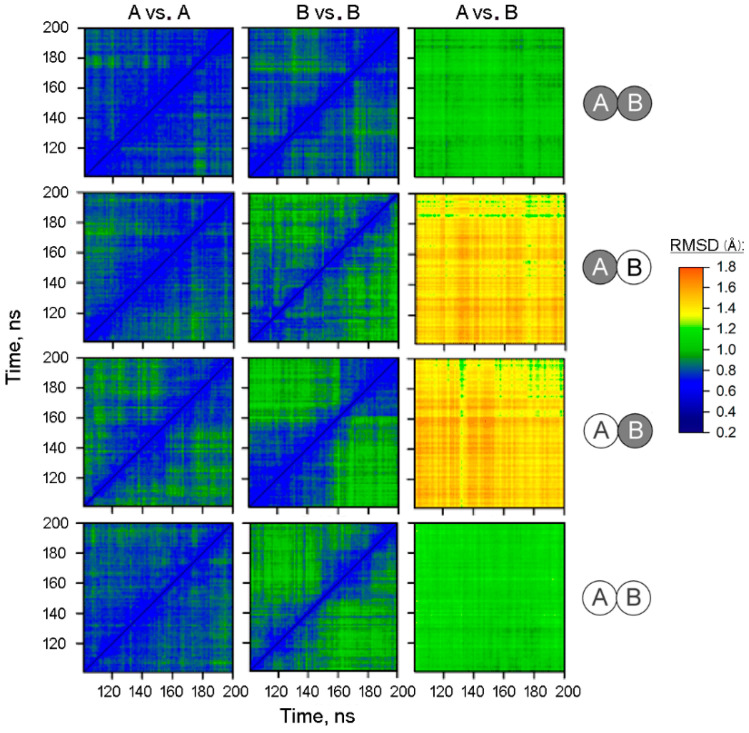
2D-RMSD analysis of the MD trajectories of the IDP complexes of Vr-mPPase of different stoichiometry. Structure snapshots were taken each 1 ns between 100 and 200 ns of the simulation. The RMSD values shown refer only to α-helix backbone atoms. The IDP content of subunits A and B in the dimer is indicated on the right; IDP-free and IDP-bound subunits are shown as open and closed circles, respectively. The top labels indicate the subunits compared. Points are colored according to the RMSD value, as shown on the color scale.

To test the modeled structures for subunit asymmetry, the full-length trajectories of subunits within the dimer were compared using a 2D-RMSD approach. In this method, one compares the structure of a subunit with the structure of the same or other subunit at each time point and calculates the RMSD value for the backbone atoms of the α-helices for each comparison. A two-dimensional plot is then constructed wherein the point color indicates the RMSD value (Figure 1). The panel columns “A vs. A” and “B vs. B” refer to comparisons within the same subunit of the dimer containing IDP in both or only one subunit(s) or in none of them, as indicated on the right side of the figure. The panels of the two left columns demonstrate diagonal symmetry, suggesting that the structure approaches equilibrium with an RMSD of 0.6–0.8 Å after 100 ns of the simulations. In contrast, the comparison of subunits A and B (the right column of panels) indicated that they evolved to different structures. This difference was characterized by the RMSD of ~1.0 Å in the IDP-free dimer, which increases to the RMSD of 1.3–1.7 Å upon IDP binding to one of the subunits. Interestingly, the fully IDP-occupied dimers demonstrated a similar degree of asymmetry as the IDP-free dimers. 

### 2.2. The Structural Elements Demonstrating Asymmetry in Vr-mPPase 

**Figure 2:**
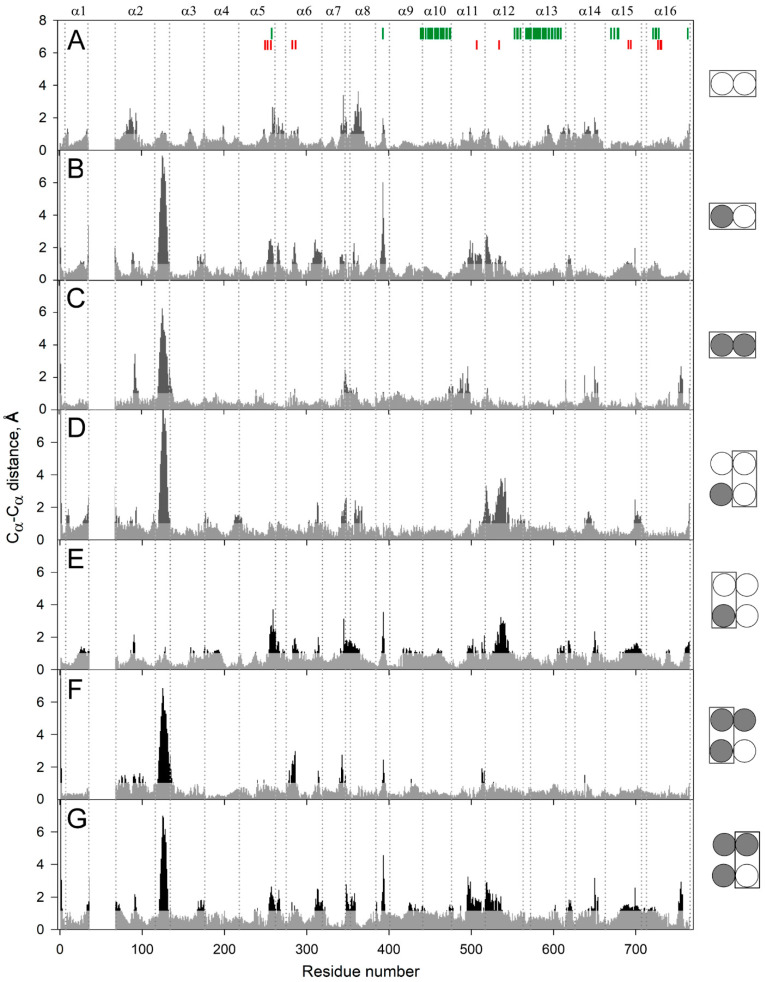
C_α_ atom displacements between superimposed MD-simulated subunit structures of the different IDP complexes of Vr-mPPase. The schemes on the right indicate the binding state of the subunits compared within or between dimer(s) in panels **A**–**G**. IDP-free and IDP-bound subunits are shown as open and closed circles, respectively; the superimposed subunits are boxed. Black areas on the profile refer to the residues whose C_α_ atoms misalign by > 1 Å in the structures compared. Vertical dotted lines separate the residues belonging to different α-helices, as indicated above in panel A. The vertical bars on the top panel mark residues belonging to the active site (red) and the subunit contact zone (green).

To localize the mobile elements in the enzyme structure, we compared the predicted structures of the subunits within the dimers containing no, one, or two IDP molecules (designated as E_2_, E_2_IDP, and E_2_IDP_2_, respectively) after averaging them over 160–200 ns of the simulations. The largest and highly mobile loop 1–2c (the loop between the helices α1 and α2; “c” marks its cytoplasmical localization), absent in the crystal structure and modeled manually, was removed from the comparison. The superpositions were created with UCSF Chimera, using the transmembrane α-helices without 1–2 terminal residues as the scaffold. Panel A in Figure 2 revealed small but seemingly significant subunit asymmetry in the resting dimer E_2_. The most notable differences between subunits were predominantly observed in the cytoplasmically-oriented protein half and encompassed helices α2, α7, and α8 and loop 5–6c (the N-termini of the helices with even numbers look into the cytoplasm) (Figure 2A). The differences in helices α13 and α14 and loop 8–9 in the two subunits were less significant.

As expected, IDP binding to one subunit further increased subunit asymmetry (Figure 2B), mainly in the luminally-oriented half of the molecule (loops 2–3 and 8–9, α-helix 6). The position of the loop 2–3 underwent the most extensive changes upon IDP binding. The C_α_ of Phe393 in loop 8–9 moved substantially in the IDP-bound subunit (Figure 2B); however, the sidechain phenyl group, a part of the subunit contact, only slightly changed its position. IDP binding also augmented asymmetry in the cytoplasmic regions of the helices α11 and α12. On the other hand, the IDP binding decreased asymmetry in the cytoplasmic parts of α7, α8, α11, and α12 and the corresponding loops. The asymmetry persisted in E_2_IDP_2_ (Figure 2C), again mainly in the loop 2–3. Three other regions with significant differences are loop 7–8c and helices α11, α14. The comparisons described above are for monomer A, but parallel superpositions for monomer B revealed similar trends in dimer asymmetry (Figure S3). 

In the next step, we compared subunits between different dimers. Surprisingly, the structure of the vacant subunit demonstrated significant differences between E_2_ and E_2_IDP (Figure 2D). Again, the most significant differences localized in the lumenal loop 2–3 and the cytoplasmic halves of helices α7, α8, and α12. Even more surprisingly, loop 2–3 structures were similar in E_2_ and the IDP-bound subunit of E_2_IDP (Figure 2E), unlike subunits within E_2_IDP (Figure 2B). These observations indicated that IDP binding affected loop 2–3 of only the neighboring subunit. In the IDP-bound subunits of E_2_IDP_2_ and E_2_IDP, the positions of loop 2–3 were different (Figure 2F), as they were in the occupied and free subunits of the same dimer (Figure 2G). 

A less striking and smaller size variation was in loop 5–6c, another functionally important element of plant mPPase structure. According to Shah et al. [19], loop 5–6c physically controls substrate entrance into the active site. Of the seven subunit pairs compared in Figure 2, the loop 5–6c structure was the same in panels C and D and, perhaps, in panel F. 

**Figure 3:**
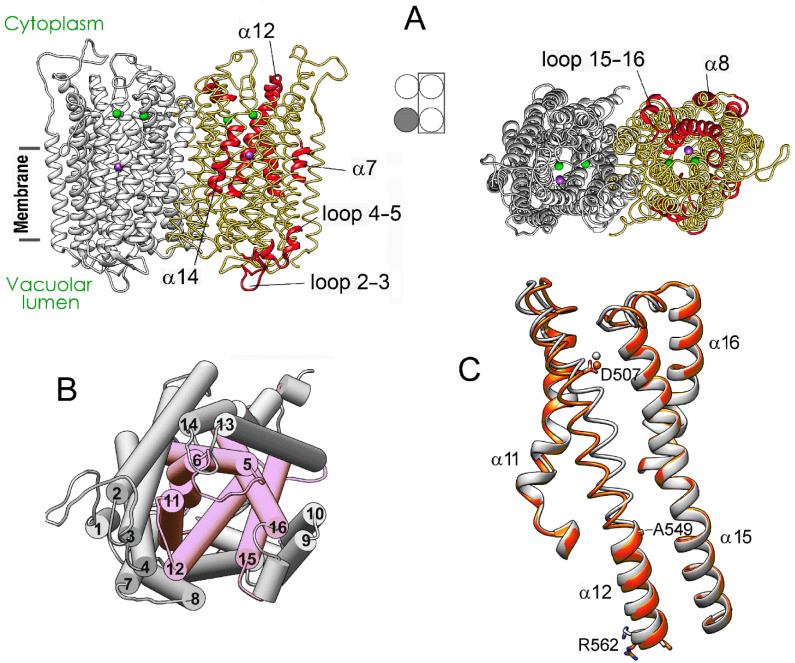
Structure changes in a subunit of the resting Vr-mPPase dimer upon IDP binding to the neighboring subunit. (**A**) The structure of the resting dimer showing the regions (colored red in one subunit) that will undergo the most significant displacements upon IDP binding to the other subunit. Subunit occupancy with IDP in the dimers compared is indicated in the middle scheme, like in Figure 2. Two bound Mg^2+^ and one K^+^ ions are shown as green and magenta spheres, respectively. (**B**) The same view on the cytoplasmic side of the left-side subunit as on the right part of panel *A*, but the helices are shown as cylinders and numbered according to Figure 2. The six helices forming the inner ring are pink. (**C**) Bending of α12 in subunit B upon IDP binding to subunit A of mPPase dimer. The panel shows a part of the superposition of the IDP-free subunits from the resting and IDP-bound dimers (grey and red, respectively) used to identify the regions marked red in panel A. Bound Mg^2+^ ion is shown as a sphere of the same color. This comparison corresponds to Figure 2D. The molecule was rotated by approximately 180° compared to the view in (**A**).

Figure 3 details, in a 3D structure, the positions of the regions demonstrating the most significant motions in a subunit upon successive IDP binding to the other subunit of the dimeric enzyme. These images correspond to the comparison of separate subunits in panel D of Figure 2. Most changes in the vacant subunit upon IDP binding to the neighboring subunit (the first binding step) occur in the peripheral regions (Figure 3A), except for α-helix 12 (α12), which belongs to the internal α-helix ring but protrudes outside in the lumenal part (Figure 3B). While the external α10 and α13 helices are the primary contributors to the subunit interface, interactions of α12 also stabilize the dimer. α-Helix 12 is significantly bent in the resting state, and the IDP binding to the neighboring subunit further bent it in a different plane, with an ~4 Å displacement in the active site region. This displacement is transferred to the lumenal part like in a lever with the pivot in the region of Ala549 located above the hydrophobic gate (Figure 3C). Interestingly, the luminal part of α12 underwent only minor changes in the IDP-bond subunit. In this context, α12 may form the pathway for subunit communication that allows sensing active site changes in the neighboring subunit. In contrast, the second binding step affected mainly the central and lumenal regions of the neighboring subunit (Figure S4). 

**Figure 4:**
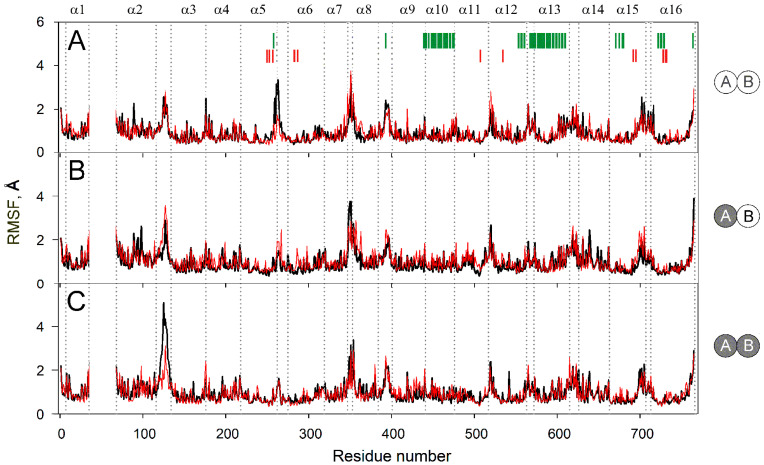
Residue sidechain fluctuations (RMSF) in the MD-simulated subunit structures of the IDP complexes of Vr-mPPase of different stoichiometry. The data were collected during 160–200 ns of the simulation. IDP-free and IDP-bound subunits within the dimer are shown as open and closed circles, respectively, on the right-side schemes for panels **A**–**C**. Black and red lines refer to subunits A and B, respectively. Other details of the structures and notations are found in the Figure 2 legend.

Figure 4 compares sidechain mobilities in dimers with different IDP content. As expected, most mobile regions are found in the loops and adjacent helix ends, mainly in the cytoplasmic part because it is larger than the lumenal one and protrudes more out of the membrane (Figure 3A). There are 8–10 mobile regions in total, and their mobilities do not change significantly upon IDP binding, except for two loops located on the opposite sides of the membrane. Subunit A occupancy adds mobility to lumenal loop 2–3 in subunit B (Figure 4A,B), while subsequent IDP binding to subunit B exerts a similar effect on loop 2–3 in subunit A (Figure 4B,C). In other words, IDP binding allosterically affects loop 2–3 behavior in the partner subunit. The IDP effects on the cytoplasmic loop 5–6c differ in that loop mobility decreases and this effect is observed in the subunit that binds IDP (Figure 4). The decreased mobility of loop 5–6c in the enzyme-substrate complexes in Figure 4 agrees with the data of Shah et al. [19] and results from the direct interaction of the IDP molecule with this loop. The effect of IDP binding on loop 2–3 is indirect because it is located on the other side of the membrane and becomes more mobile upon IDP binding. The increased loop 2–3 mobility may compensate for the unfavorable change in entropy caused by substrate binding and fixing the loop 5–6c position. 

### 2.3. Loop 2–3 Structures in Various Vr-mPPase Complexes

**Figure 5:**
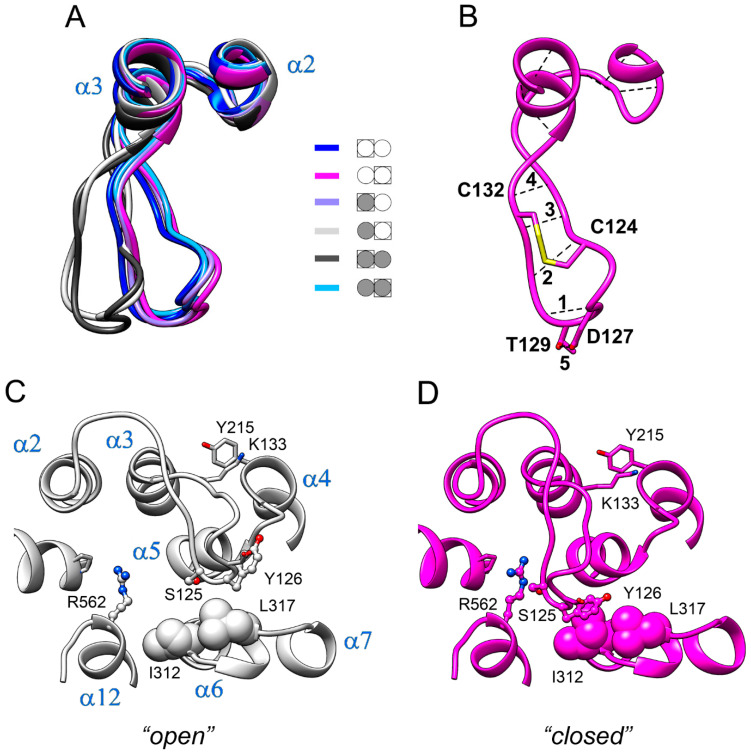
Two conformations of loop 2–3 observed in Vr-mPPase subunits. (**A**) The loop 2–3 part of the superposition of the six types of subunits found in Vr-mPPase dimers containing from zero to two bound IDP molecules. The color scheme shown defines dimer occupancy with IDP (closed circle) and the subunit selected for comparison (boxed). Molecule orientation is different from Figure 3A. (**B**) The intraloop H-bonds (dashed lines) and covalent S-S bond between two cysteine residues (thick yellow line). The H-bonds are numbered. (**C,D**) A more detailed view from the opposite side of the two conformations of loop 2–3 with nearby amino acid residues. A ball-and-stick or space-filling representation shows the sidechains of selected amino acid residues (marked with a one-letter code). α-Helices are labelled in panel C.

Loop 2–3 demonstrated the largest displacements between different structures in Figure 2. As subunit structures are different within both the IDP-free and fully-occupied dimers, there are six different subunit structures in total. Figure 5A shows the loop 2–3 part of the superposition of all six structures. Interestingly, the loop adopted two distinct conformations, which we will contingently call “open” and “closed”. The “open” conformation was found in the IDP-free subunit of E_2_IDP and in one subunit of E_2_IDP_2_. These identifications are in accord with the data in Figure 2, where the loop 2–3 peaks indicate the difference in the loop conformations in the compared subunits. 

The structure of loop 2–3 explains its “rigid-body”-like behavior. The loop contains two proline residues (Pro121 and Pro134) and is cross-linked by the Cys124–Cys132 disulfide bond (Figure 5B). Moreover, several intraloop H-bonds (Figure 5B) are formed in both types of loop structures (Table S1). On the other hand, the hinge part of the loop contains two glycine residues (loop Gly116 and α2 Gly112), which allow loop displacement as a rigid body. Loop residues also form important hydrophobic contacts, which center around Tyr126. In the “closed” conformation, the aromatic ring of Tyr126 contacts Ile312 and Leu317 of loop 6–7 (panel C). In the alternative (“open”) conformation, Tyr126 moves out of its contact with Ile312. The hydrophobic interaction of Lys133 with Tyr215 of the α4 terminus at the loop base also stabilizes the loop shape; this interaction is preserved in both loop conformations. The aromatic moiety of Tyr126 thus slides on the hydrophobic surface formed by Ile312 and Leu317 when loop 2–3 swings between its two conformations.

### 2.4. The Interactions Stabilizing Loop 2–3 Positions

**Figure 6:**
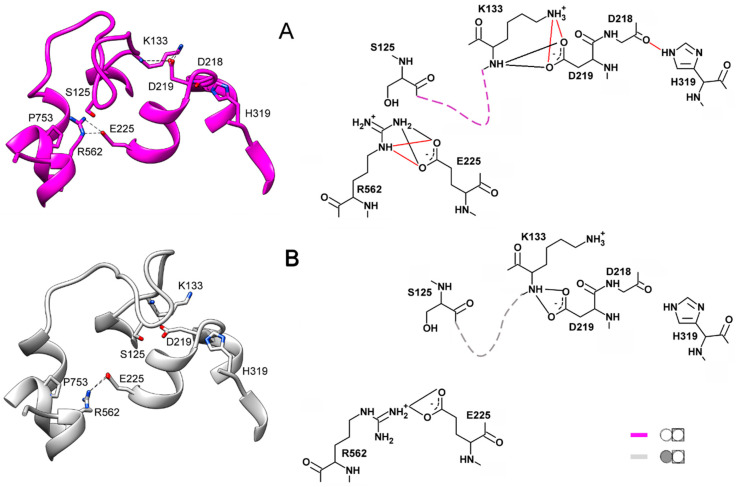
External H-bonds stabilizing the two conformations of loop 2–3 in IDP-free subunits. Loop 2–3 is shown schematically by dashed lines on the right images. The main colors correspond to the color scheme defined in Figure 5. (**A**) Subunit B of IDP-free dimer. (**B**) IDP-free subunit B; its neighboring subunit contains IDP. The H-bonds depicted in red in the right part of panel A are those disrupted in the structure shown in panel B.

Two important interactions outside the loop additionally stabilize the loop conformations. One is the H-bonds connecting loop Lys133 to Asp219 of a short (four residues) loop 4–5. In the closed-loop conformation, the Asp219 sidechain carboxylate interacts with both the mainchain imido and sidechain amino groups of Lys133, and the salt bridge Asp218···His319 buttresses these interactions (Figure 6A). Only the H-bonds between Asp219 and the Lys133 mainchain NH are retained in the open-loop conformation (Figure 6B). 

The interaction of Arg562 of α12 and Glu225 of α5 is another crucial factor contributing to the selection of the loop 2–3 conformation. This interaction involves two Arg sidechain nitrogens in the closed-loop conformation and only one in the open one (Figure 6). Changes in the H-bonding are naturally associated with changes in distances and drive loop motions. We believe that Arg562, localized in the luminal part of α12, is the critical element determining loop 2–3 conformation. This notion is consistent with the correlation between the loop 2–3 conformation and the degree of α12 deformation in the luminal parts of subunits upon IDP binding to one of them and is supported by the mutagenesis data (see below). Helices α5 and α6 are other crucial communication elements because they form a movable active site “lid”, loop 5–6c, ref. [11] and relay its motion to Asp218, Asp219, and Glu225, which are Arg562 partners. 

The above conclusions on the H-bonding in different loop 2–3 states are based on the H-bonds’ frequencies in the multiple IDP-free structures generated during the MD simulations. For instance, Asp219 interacts with Lys in almost all (94%) snapshots of IDP-free neighbors of IDP-bound subunits, and this interaction is exclusively through the Lys backbone N (Table S2). If no IDP is bound in the neighboring subunit, both Lys133–Asp219 interactions are observed in similar occurrences. In total, the two residues interact in most (65%) snapshots, of which 13% demonstrate double H-bonds formed through both Lys133 N atoms. Like the H-bonds in Table S1, the Lys133–Asp219 interaction is preserved in both loop 2–3 conformations, although it is appreciably modified in the closed conformation. 

The same considerations apply to the Glu225–Arg562 interaction (Table S2), but the difference is that the H-bonds rearrange instead of being completely broken. The interaction involves the Arg562 NHω atoms in one case (the IDP-free partner of the IDP-bound subunit) but the NHω and Nε atoms in all other cases. This H-bond rearrangement allows loop displacement. 

### 2.5. Loop 2–3 Conservation in mPPases

**Figure 7:**
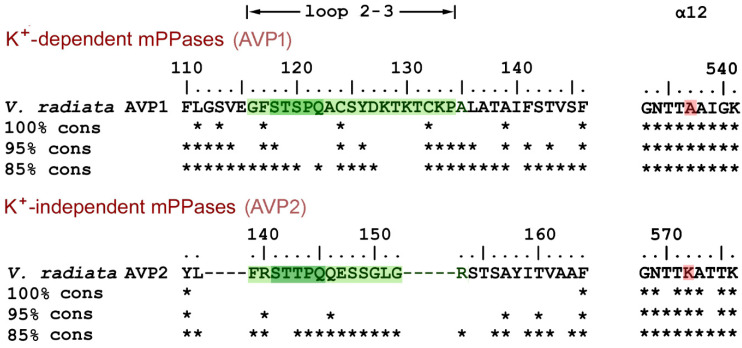
Alignment of loop 2–3 sequences in eukaryotic mPPases. The upper line on each part shows Vr-mPPase sequence, and the other lines indicate by asterisks the residues conserved in 100, 95, and 85% of the indicated mPPase subgroup sequences. Loop 2–3 sequences are highlighted in green. The gaps shown are found in all members of the AVP2 subgroup. Parts of the α12 sequences shown on the right contain the K^+^ dependence signature residue (highlighted in red).

mPPases are found in bacteria, archaea, protists, and plants, but not in other eukaryotes. A blast [20] search in the KEGG database using Vr-mPPase sequence as the query yielded 2436 nonredundant mPPase sequences. Clustal X [21] alignment classified them into two groups, based on loop 2–3 sequences. In the group comprising all eukaryotic mPPases, loop 2–3 is of appreciable length (14–19 residues), whereas it is reduced to the minimum length needed to connect two helices in the prokaryotic mPPase group. The eukaryotic mPPases are further divided into two subgroups with different loop 2–3 sequences, which are highly conserved within the subgroups (Figure 7) but resemble each other only in the five-residue fragment STS(T)PQ. The members of the larger subgroup are classified as vacuolar K^+^-dependent enzymes (AVP1) based on the presence of Ala, not Lys, in the K^+^ dependence signature residue position (537 in *V. radiata* AVP1 mPPase) [22] (Figure 7). This subgroup contains only Viridiplantae (green plant) mPPases (327 sequences from 91 species) but no mPPases of other phyla. The smaller subgroup (AVP2 mPPases; 130 sequences from 101 species, primarily Viridiplantae) comprises K^+^-independent enzymes [23] with apparently non-vacuolar localization in plants [24]. *V. radiata* and other plants contain both AVP1 and AVP2 mPPases. 

The Loop 2–3 structure and its associated elements are highly conserved in the plant AVP1 mPPases. Two Cys residues of loop 2–3 are absolutely conserved, and Tyr126 is highly conserved (>95%). Moreover, one of Tyr126 interacting partners, Ile312, is absolutely conserved (not shown), and the other, Leu317 in Vr-mPPase, is Ile, a similar hydrophobic residue, in most sequences. Two other residues interacting with loop 2–3, Arg562 from α12 and Pro753 from α-helix 16, are also found in >95% K^+^-dependent mPPases. 

### 2.6. Mutations in Loop 2–3 and Its Interacting Partners

Asaoka et al. reported the effects of substituting three loop 2–3 residues (Cys124, Tyr126, and Cys132) by Ala on Vr-mPPase activity in PP_i_ hydrolysis and H^+^ transport [25]. The Cys124/Ala substitution increased the hydrolytic activity by 90% and decreased the transport activity by 25%, resulting in a less coupled enzyme. The effects of other substitutions were less significant. These activity measurements were performed at the substrate concentration (50 µM Mg_2_PP_i_, calculated from the 1 mM total PP_i_ and MgCl_2_ used by Asaoka et al.) [25], permitting saturation of only one active site in the dimer [26]. The three-dimensional structures of the mutant proteins are unknown. We introduced identical virtual mutations into the Vr-mPPase structure and predicted the structures of the mutant proteins by MD simulations as described above for the wild-type enzyme. We sought to determine how the mutations change the conformation of loop 2–3 in both subunits of the relevant E_2_IDP complex, to which the data of Asaoka et al. [25] refer. Surprisingly, we found that the intra-loop H-bonds listed in Table S1 are retained in the IDP-free and IDP-bound subunits of the mutant proteins (Table S3). The only exception was the IDP-bound subunit of the Cys132Ala variant, in which the Cys124···Lys130 H-bond was not formed. 

As shown above (Figure 5A), loop 2–3 adopts ‘open’ or ‘closed’ conformation in wild-type Vr-mPPase, depending on the IDP content of the dimer. None of the mutant mPPases resembled the wild-type enzyme in this aspect (Figure S5). In only the Cys124Ala variant, loop 2–3 adopted a similar “closed” conformation in the IDP-bound subunit (Figure S5A). In the other variants, loop 2–3 conformation was intermediate between “open” and “closed” in this subunit. In the neighboring, IDP-free subunit, the loop adopted a “closed” conformation in all mPPase variants, contrasting its “open” conformation in the wild-type enzyme (Figure S5B).

**Figure 8:**
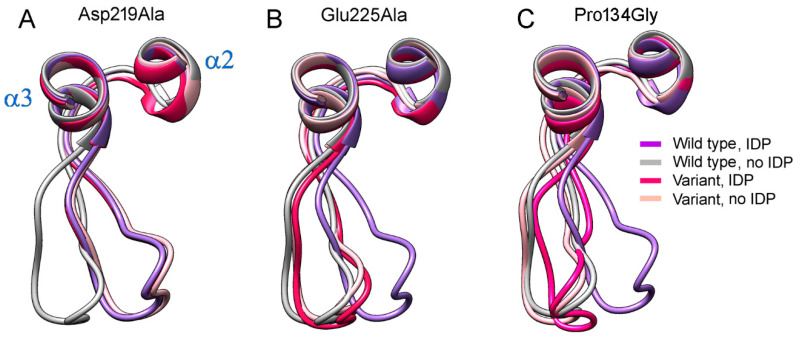
The effects of three amino acid substitutions on loop 2–3 conformation in dimeric Vr-mPPase containing IDP in one subunit. (**A**) The Asp219Ala variant. (**B**) The Glu225Ala variant. (**C**) The Pro134Gly variant. Each panel shows wild- type mPPase data for comparison. The color scheme defines the Vr-mPPase type and subunit occupancy by IDP.

The highly conserved Pro134 residue (Figure 7) makes a rigid junction between loop 2–3 and α-helix 3. When Pro134 was virtually replaced by the more permissive Gly, the loop adopted similar conformations in both subunits of E_2_IDP (Figure 8C), which were close to the “open” conformation observed in the vacant subunit of the corresponding wild-type enzyme complex. This finding indicated that loop 2–3 rigidity at its C-terminus is required for adopting the “open” loop conformation and that it is more strained than the “closed” one.

Two non-loop acidic residues, Asp219 and Glu225, are involved in stabilizing the loop 2–3 conformations (Figure 6). Earlier measurements indicated a decreased transport activity upon Glu225 replacement by Ala [27,28]. Moreover, a similar substitution of the Glu225 partner, Arg562, decreased the coupling ratio by 30% [29]. Asp219, Glu225, and Arg562 are absolutely conserved in plant AVP1 mPPases and absent in other mPPase lineages. Tsai et al. [27] suggested that the Glu225···Arg562 salt-bridge ‘might act as a latch to regulate proton release through the exit channel in plant mPPases’. The effect of Asp219 replacement in any mPPase has been unknown, but the substitution of the neighboring Asp218 in Vr-mPPase also decreased the coupling ratio more than two-fold. As these functional characterizations were performed under conditions allowing binding of only one substrate molecule per dimer, we predicted the structures of the Asp219Ala, Glu225Ala, and the double Asp219Ala/Glu 225Ala Vr-mPPase variants containing IDP in only one subunit. 

The predicted structures of the Asp219- and Glu225-substituted variants indicated that any single substitution fixed loop 2–3 in one conformation in both subunits irrespective of their IDP content (Figure 8). The conformation was “closed” in the Asp219Ala but “open” in the Glu225Ala variant. Thus, the single substitutions canceled the dependence of loop conformation on active-site occupancy by IDP. In the double Asp219Ala/Glu225Ala variant, the loop did not tend to occupy a fixed “closed” or “open” position but stochastically fluctuated between them during the 200 ns of the simulation. In both variant types, the Glu225Ala substitution forced the Arg562 sidechain to move inside the exit channel, seemingly hampering proton transport through it. The loop structure and “rigid body”-like behavior were not affected by the substitutions.

### 2.7. Water Accessibility in Structures with Different Loop 2-3 Conformations

**Figure 9:**
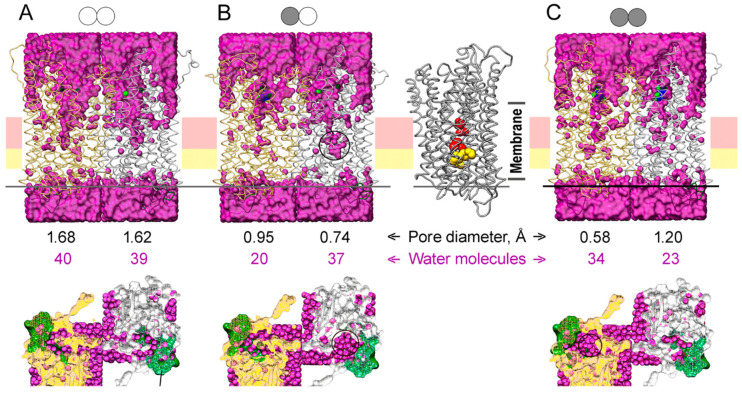
The water content of the MD-simulated structures of the IDP complexes of Vr-mPPase of different stoichiometry in a membrane/aqueous environment: (**A**) the IDP-free enzyme; (**B**) the enzyme with one IDP-bound subunit; (**C**) the fully IDP-bound enzyme. The top panels show the view along the membrane plane. Water oxygens are magenta circles, and two Vr-mPPase subunits are depicted in a ribbon mode in gold and grey colors. The schemes above indicate a dimer binding state; IDP-free and IDP-bound subunits are shown, respectively, by open and closed circles. The bottom panels present the top view of the exit channel cross-section, whose position is indicated by the horizontal line on the top panels. The black circles on the three panels mark the regions with enriched water content compared to the IDP-free dimer. The right part of panel *B* shows the IDP-bound subunit with the residues forming the ionic channel (Arg242, Asp294, Lys742, and Glu301; red) and the hydrophobic gate (Leu232, Ala305, Leu555, and Val746; yellow) in a space-filling representation. The positions of these elements on the upper panels are indicated by horizontal pink and yellow stripes. The two rows of numbers between the top and bottom panels show the minimal pore diameter of the ion-conducting channel at the hydrophobic gate and the combined number of the water molecule in the ionic channel and the hydrophobic gate.

Water molecules were positioned in the protein structures of different binding states using a GIST function of CPPTRAJ and Placevent (Figure 9). The water content of the active site cavity and channel entrance was higher in all simulated IDP-free subunit structures (Figure 9). IDP binding to one subunit caused the closure of its active site and constriction of ion conductance channel at its ionic part formed by Arg242, Asp294, Lys742, and Glu301 and the hydrophobic part formed by Leu232, Ala305, Leu555, and Val 746 [8]. As a result, the water content of this part of the ion-conducting channel decreased two-fold (compare Figure 9A and 9B). The water content of the other subunit did not change significantly, but the water was displaced closer to the gate. IDP binding to the second subunit decreased its water content but increased the water content of the neighboring subunit, reversing subunit asymmetry in this respect. The water molecules found in the crystal structure of the E_2_IDP_2_ complex of Vr-mPPase [8] (25 molecules in one and 22 in the other subunit) represent only bound water molecules. More water molecules (marked by circles on Figure 9 bottom panels) were seen at the cross-section above loop 2–3 in the subunits whose loop conformation is “open” according to Figure 5A than in the “closed”-loop subunits.

To determine the consequence of water displacement in the IDP-free subunit, we performed pore size analysis in different subunit types in Figure 9 with PoreWalker [30], as used previously for the crystal structure [27]. The minimal values of the pore diameter of the ion-conducting channel around the hydrophobic gate (at Leu555) are shown in Figure 9. Subunit pore diameters were similar (1.62–1.68 Å) in the IDF-free dimer (Figure 9A). However, pore diameters differed in the E_2_IDP complex and more in the E_2_IDP_2_ complex (Figure 9B,C), consistent with dimer asymmetry. Remarkably, the pore diameter decreased more significantly in the IDP-free than IDP-bound subunit in E_2_IDP (Figure 9B), presumably because the displaced interior water at both gate sides enhanced the hydrophobic effect. Noteworthy, our estimates of the pore size in E_2_IDP_2_ are smaller than in the corresponding crystal structure (2.1 Å) [27]. 

## 3. Discussion

Catalytic asymmetry (active-site interdependence) is an inherent property of mPPases, including plant AVP1 enzymes [14,15,16,31], rather than the effect of the membrane [15]. Here we sought to determine the structural basis of the substrate-binding asymmetry using a computational method with the experimentally determined IDP-complexed Vr-mPPase structure as the starting point. An essential advantage of the MD approach is that it allows prediction of the dimer structure with only one active site occupied by substrate, not easily obtainable experimentally.

### 3.1. Structural Asymmetry in Vr-mPPase Dimer—A Myth or Reality?

Several crystal structures of Vr-mPPase and Tm-mPPase complexed with IDP or P_i_ and one structure of free Tm-mPPase have been published [18] and are all described as dimers of very similar subunits, with RMSD of 0.32–0.58 Å for Cα atoms [8,9,11]. Noteworthy, the phosphorus ligands occupy both active sites in these structures because mPPase was crystallized at very high ligand concentrations. The RMSD value comparing subunit structures in the MD-generated E_2_IDP_2_ complex of Vr-mPPase was ~1 Å, three times greater than in the corresponding crystal structure [8], suggesting some effect of ordered packing in the crystals.

Why was asymmetry not detected in the crystal structures? Apart from the effect of the crystal lattice, a trivial explanation, still consistent with the functional asymmetry, could have been that these structures are indeed symmetrical. One could have speculated that binding the first substrate molecule to an initially symmetrical dimer induced structural changes in the partner subunit (hence increased *K*_m_ for the second substrate molecule) and that the binding of the second substrate molecule restored symmetry by changing both subunit structures (hence, decreased *k*_cat_). However, the MD data are inconsistent with this scenario. They revealed a small but significant difference in the subunit structures in the resting dimer (Figure 2A) and a larger difference in the fully-occupied dimer (Figure 2C). These observations suggest that the structural asymmetry is an inherent property of mPPase, which is only modulated by substrate binding.

How significant should structural asymmetry be to invoke the catalytic asymmetry? Clearly, it is not the size of the structural change that matters but the energy required for this change. Even a tiny conformational change may cause appreciable strain, consuming and, consequently, storing palpable energy. One knows from chemistry that the separation of reacting particles by only 1 Å may significantly affect the reaction rate. Although large conformational changes are not infrequent in multidomain proteins, they are unlikely in a compact one-domain protein, like mPPase, because of high energy cost. On the other hand, a tiny change may go undetected by the structure refinement program, which may prefer to treat a slight difference as a diffraction data scatter.

**Figure 10:**
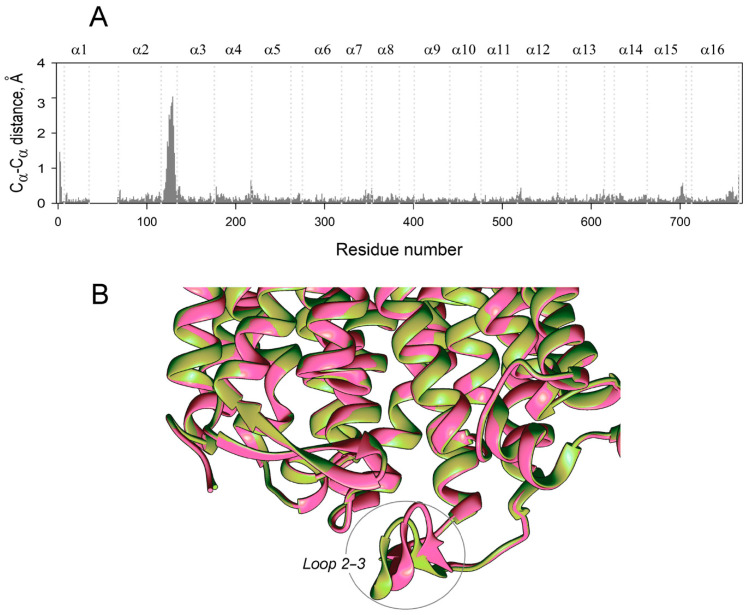
Subunit asymmetry in the published crystal structure of Vr-mPPase containing IDP in both subunits (PDB ID: 4A01) [8]. (**A**) C_α_ atom displacements between superimposed subunit structures. Other notations are as in Figure 2. (**B**) Part of the subunit superposition in the loop 2–3 region. Two subunits are shown in different colors.

Finally, the usefulness of RMSD for comparing subunit structures is questionable because even large displacements of few amino acid residues do not contribute significantly to the RMSD value, which refers to the whole molecule. Indeed, re-examining the IDP-bound Vr-mPPase crystal structure, classified as symmetrical based on the RMSD value of 0.32 Å [8], indicated an uneven distribution of residue displacements along the polypeptide, with a prominent peak in the region of loop 2–3 (Figure 10A). Examination of the loop structure revealed that the loop conformations in the subunits were different, contrasting the rest of the molecule (Figure 10B), and resembled the two conformations found in the MD-simulated structures (Figure 2C). This finding supports the inference from the MD data that the E_2_IDP_2_ complex is asymmetrical in the loop 2–3 region. While this asymmetry can be characterized as local in the crystal structure [32,33], it extends to other regions in the MD-simulated structures (Figure 2C), which are expected to resemble the solution structures better.

In the resting enzyme, loop 2–3 adopts similar conformations in two subunits according to the MD data (Figure 2A and Figure 5A). However, the subunit structures demonstrate modest differences in other regions, suggesting significant pre-existing asymmetry. Different mobilities of loops 2–3 and 5–6 in subunits A and B in the resting and fully-occupied dimers (Figure 4A,C) support this assertion. 

### 3.2. Loop 2–3 and Other Asymmetry-Related Elements in mPPase

Our data indicate that loop 2–3 demonstrates the most considerable structural difference between mPPase subunits, but this difference emerges only when IDP (substrate) binds to at least one subunit. As the active site and loop are located on opposite sides of the vacuolar membrane, a structural link should connect them. Moreover, and most intriguingly, this link extends to the other subunit because IDP binding affects loop 2–3 conformation not in the same but the other, vacant subunit. Our data identify α12 as the central element interconnecting the active sites and loops 2–3 in the enzyme dimer because it participates in the active site and subunit interface and contains Arg562, which controls loop conformation. This inference is supported by the findings of Vidilaseris et al. [17], who used X-ray crystallography to investigate the asymmetry in Tm-mPPase complexed with ATC. This inhibitor binds to loops 6–7 (its β-sheet element), 8–9, and 12–13 in the luminal part of only one subunit of Tm-mPPase, which has a very short loop 2–3. 

One can envisage the following structure mechanics following IDP (substrate) binding. IDP binding to subunit A causes active site constriction, resulting in downward (to the lumen) shifts of the inner α-helices, including α12. These motions are propagated to subunit B through the rigid interface formed by α-helices 10, 12, 13, and 15. As a result, α12 is bent and displaced upward (to the cytoplasm) in subunit B (this motion is more clearly seen in Figure S3B that mirrors Figure 2D). Accordingly, Arg562 found in α12 is shifted, and loop 2–3 changes its position from relaxed “closed” to strained “open”. The strain in loop 2–3 conformation is made possible by its “rigid-body”-like structure and the presence of proline (residue 134) in the loop junction to α-helix 3. Among other things, α12 displacement confers catalytic activity to subunit A by allowing two Asp residues (287 and 731) to activate the nucleophilic water molecule [11] but distorts the active site in subunit B. Consequently, substrate forms fewer contacts with this active site, resulting in a markedly increased *K*_m_ value for the second substrate molecule, inability to constrict active site and change, in a crosswise manner, loop 2–3 conformation in subunit A. 

The crystal structures suggested that intersubunit communication may additionally involve an H-bonded triad of residues (Glu212–Arg578–Ile399’ in Tm-mPPase and Glu263–Arg609–Arg441’ in Vr-mPPase crystals) that belong to helices 5, 13 of one subunit, and α-helix 10 of the other subunit [11]. The triad may link the motion of the inner ring upon substrate binding to the outer ring (subunit interface) and Ile399/Arg441 of the other subunit. This triple symmetry-related interaction is abolished in the IDP-lacking crystal structures [11]. In the simulated structures, the triad was similarly not formed in the IDP-free subunits but was, at least partially, formed in the IDP-bound subunits to which the first two residues of the triad belong (Table S4). This observation provides support for the MD-simulated structures and reveals another factor of structural asymmetry. Noteworthy, the first two residues of the triad interact via sidechains and are absolutely conserved in all mPPases. The last residue, belonging to the other subunit, interacts via mainchain oxygen and is not consequently conserved (Arg441 in Vr-mPPase and Ile399 in Tm-mPPase).

### 3.3. A Possible Role of Asymmetry in the Coupling Mechanism of mPPase

We have earlier speculated that, despite using a different, direct-coupling mechanism, mPPase resembles F-ATPase in transiently converting the energy of substrate binding into a conformational strain of the other subunit [12,15]. We further assumed that the stored energy might be back-used to finish the catalytic cycle [15]. The relevant part of a hypothetical coupling mechanism was formulated in the following way [15]: ‘(a) substrate binds to active site A, resulting in a conformational change (site lid closure) that is propagated to active site B in the other subunit; (b) the substrate is attacked by the activated water molecule located at the entrance to the ion-conducting channel. This reaction yields two phosphate molecules that remain bound in the active site and a proton, which creates high local acidity; (c) the proton moves via a water wire to the other side of the membrane down the locally favorable proton potential gradient; (d) the loop lid opens because its weaker interactions with the two phosphates cannot offset the conformational strain, and the phosphates leave the active site. The enzyme dimer thus performs asymmetrically—one subunit carries out PP_i_ hydrolysis and ion transport while the other acts as a transient energy store.’ These speculations were based on the kinetic data showing that substrate or substrate analog binding to one active site impedes the binding and catalytic abilities of the other active site of homodimeric mPPase [14,15]. The alternative model of mPPase catalysis [17] assumes that the energy released in the substrate-binding step is immediately used in the same subunit to drive H^+^ transport before PP_i_ hydrolysis. However, as we pointed out elsewhere [15], this model disagrees with the electrometric data [11,19].

The new data provide a structural basis for our proposed mechanism in its part referring to substrate binding. By detailing the substrate-induced changes in the neighboring subunit, our data define the core of the mechanism by which this partner subunit transiently accumulates the energy released upon substrate binding. The primary strain is likely accumulated in α-helix 12 and loop 2–3, which demonstrated the most considerable changes in structure upon IDP binding to the neighboring subunit (Figure 2D). As α12 contains an active-site Mg^2+^ ligand (Asp507), which interacts with PP_i_, α12 displacement may explain the increased *K*_m_ for the second substrate molecule. Arg562, which is on the other side of α12, links α12 and loop 2–3 motions. Of course, one should keep in mind that the MD information refers to a short period (0.2 µs in our case) compared with the time required for the complete catalytic cycle (0.1 s), which may involve slower structure rearrangements than one can observe in MD. 

Both enzymatic and transport activities of mPPase decrease when the second substrate molecule binds to the dimer [13,14,15,16]. These effects mean that the catalytic efficiency of either active site in the fully occupied dimer is less than in the dimer with only one active site occupied. As subunit structures differ a lot in the fully occupied dimer, its two active sites likely perform differently, or, in the ultimate case, only one of them is functional. Different pore sizes in the E_2_IDP_2_ subunits support this conclusion. Noteworthy, one subunit of E_2_IDP_2_ has the smallest pore size, which may hamper the proton transfer step in this subunit and make it inactive in the complex with PP_i_. 

Because the resting dimer is asymmetrical, its subunits may permanently have unequal IDP and substrate binding probabilities unless they undergo a rapid flip-flop. The presence of loop 2–3 peaks on all panels of Figure S3 suggests that such a flip-flop does occur. On the other hand, lower heights of the loop 2–3 peaks in Figure S3 (particularly on panels A and C compared to panels B and F in Figure 2) may mean that the flip-flop requires more than 200 ns for completion.

### 3.4. Loop 2–3 Role in mPPase Asymmetry and Functioning

The largest IDP-induced motions of loop 2–3 in Vr-mPPase are a consequence rather than the cause of the asymmetry. Two arguments support this deduction. First, this loop is very short, practically absent in other, non-AVP1 mPPases, which nevertheless demonstrate catalytic asymmetry [13,14,15]. Furthermore, the loop is not essential for the hydrolysis or transport reaction catalyzed by all mPPases regardless of the loop length. What is the role of loop 2–3, and why is it found only in plant vacuolar mPPases? 

A plant vacuole is a cell compartment with a highly acidic interior (lumen). The acidity is maintained, besides other means [34], by two H^+^ pumps—V-ATPase (a close analog of F-ATPase) and mPPase [35,36]. Their work and lumen acidity should be regulated [37], and loop 2–3 is a likely candidate for this role in mPPase. Three lines of evidence support this hypothesis. First, our data show a strong link between the active site and loop 2–3, suggesting a possibility to affect active site performance and H^+^ transport via the loop. Second, loop 2–3 conformation is controlled by two ionizable amino acid residues, Asp219 and Glu225 (and, possibly, Asp218). Assuming that the alanine replacement mimics protonation by removing charge, one can suggest that H^+^ binding by these residues will cause similar effects on loop 2–3 conformation. Finally, loop 2–3 sequence is highly conserved in vacuolar mPPases; furthermore, its interacting partners (Asp219, Glu225, and Arg562) are absolutely conserved in vacuolar mPPases but are not found in other mPPase types. 

Although the effect of loop 2–3 conformation on the active site and, hence, the H^+^-pumping activity via helix α12 is possible, a modification of the H^+^ transport pathway seems more likely. As the PP_i_ hydrolysis and H^+^ transport reactions are coupled, the effect on any of them will affect both. Vidilaseris et al. [17] similarly explained the inhibition of Tm-PPase by the synthetic compound ATC [17,38], which binds to the corresponding periplasmic side of Tm-PPase, by creating a hydrophobic clamp at the gate. One can further speculate that such a clamp would be forcing the hydrolysis-generated proton to find a way to the cytoplasm instead of crossing the membrane, such as is postulated in the hypothetical mechanism of Na^+^-transporting mPPases [4,12]. Noteworthy, Tsai et al. [27] pointed out that the loop-dependent salt-bridge Glu225–Arg562, might act as a latch to regulate proton release. Alternatively, loop 2–3 may physically block the exit channel when being in the “closed” conformation because of protonation of the loop carboxylates. 

Because the free energy change associated with PP_i_ hydrolysis is considerably less than for ATP hydrolysis, the former reaction can be reversed in cell. In other words, mPPase can catalyze PP_i_ synthesis driven by the proton concentration gradient. The current view is that vacuolar mPPase does not act as a unidirectional H^+^ pump-like V-ATPase but maintains a cytoplasmic PP_i_ level by hydrolyzing or synthesizing it depending on the cell status [39,40]. PP_i_ is a vital regulator and alternative energy source present in relatively high concentrations in plant cell cytosol [7,41]. By sensing PP_i_ in the cytoplasm and H^+^ in the lumen, loop 2–3 may work as a “clutch” that determines the direction in which mPPase works. This idea does not violate the thermodynamics laws because switching the enzyme from one activity to the opposite (i.e., changing loop conformation) requires energy, which is provided from outside the enzyme. When the PP_i_ concentration is high, mPPase pumps H^+^, thereby decreasing the PP_i_ concentration and acidifying the lumen. To prevent its excessive acidification by V-ATPase, mPPase can catalyze a backflow of H^+^ coupled with PP_i_ synthesis. In this capacity, mPPase may cooperate with soluble PPase, present in the cytosol, to maintain a cytosolic PP_i_ level [42]. Loop 2-3 may thus determine the interplay between the hydrolytic and synthetic activities of mPPase. 

## 4. Methods

### 4.1. Structure Preparation for Calculations

Molecular dynamics (MD) simulations were carried out with dimeric Vr-mPPase (PDB ID: 4A01), whose crystal structure with bound IDP, 5 Mg^2+^, and 1 K^+^ ions in each active site was previously determined at a resolution of 2.35 Å [8]. The large loop connecting α-helices 1 and 2 (loop 1–2c, residues 42–66; for convenience, we mark all cytoplasmically-oriented loops with the letter “c”), not resolved in the crystal structure [8], was modeled with MODELLER [43]. Virtual mutations were introduced with UCSF Chimera [44].

The resulting structures were placed, using CHARMM-GUI [45], into a lipid bilayer formed by 1-palmitoyl-2-oleoyl-*sn*-glycero-3-phosphocholine and 1-palmitoyl-2-oleoyl-*sn*-glycero-3-phosphoethanolamine at a ratio of 1:1 and solvated by water molecules. The water layer thickness was 25 Å on each membrane side. K^+^ and Cl^-^ ions were added to mimic 100 mM KCl solution. Amber 14 force fields were used for structure parametrization: ff14SB for protein, water, and ions; lipid14 for the lipid bilayer; GAFF for IDP [46]. Following the structure of the mPPase-bound IDP [8], the О-Р-О and N-P-O angles in the IDP were decreased by 4° and 3°, respectively, compared to that of non-bound Mg_2_IDP [47]. The harmonic force constants for angles and the torsion barrier for the rotation around the P-N bond in IDP were increased to account for the distortion in the bound IDP structure and ensure complex stability [48]. Charges on the IDP molecule were calculated from quantum mechanics using an AM1-bcc force field for ionic compounds. Where indicated, the phosphorus ligand, K^+^ ion, and 3 Mg^2+^ ions were removed from one or both active sites, leaving only 2 Mg^2+^ ions; empty space was filled with water molecules. 

### 4.2. Molecular Dynamics Simulations

Calculations were performed as independent duplicate runs on a graphical station with 4 NVIDIA GeForce GTX Titan X graphics cards, operated under Ubuntu 14 with a CUDA 6.5 toolkit package. Energy minimization, heating to 310 K (37 °C), and equilibration of the structures were performed using the SANDER and PMEMD.CUDA programs of the AMBER 14 software suit. Restrains on the atoms, including the IDP, Mg^2+,^ and K^+^ ions, and 18 molecules of the crystallization water located within 5 Å from the phosphate ligand, were removed in 6 steps in strict accordance with CHARMM-GUI recommendations. The temperature was kept constant using a Langevin thermostat, and the pressure (1.0 bar) was kept constant using a Berendsen barostat.

Postprocessing trajectories were analyzed with the program CPPTRAJ of the AMBER 14 suit, and VMD [49] was used to visualize the atomic coordinate trajectories from AMBER 14 over time. The structures averaged from each 30th of the 4000 snapshots obtained between 160 and 200 ns of the MD simulations were used as the final simulated structures. 

The water distribution in the protein molecule was derived from the trajectories obtained between 160 and 200 ns of the MD simulations (each 10th of the 4000 snapshots) using GIST, as implemented in CPPTRAJ of the AMBER 14 suit. The grid size was 0.5 × 0.5 × 0.5 Å; the volume covered (35 × 35 × 90 Å) included all inner α-helices and loops (except for loop 1–2c) and, partially, outer α-helices. GIST calculated water occupancy within each voxel during 40 ns. The output file for oxygen atoms was converted into a pdb file with solvent coordinates using Placevent [50] and further analyzed with UCSF Chimera. The latter software was also used to draw structures.

## 5. Conclusions

The structural aspects of the functional asymmetry and overall transport mechanism of homodimeric mPPase are still poorly understood. The principal outcome of the current study consists of predicting the enzyme’s structure with only one active site occupied by a very close substrate analog, the structure not easily accessible by crystallography. Such a complex with the substrate PP_i_ prevails at its physiological concentrations and determines mPPase functioning in vivo. The MD-generated structures, which are expected to resemble solution structures better than the crystal ones, demonstrated structural differences between subunits in the resting enzyme and more significant differences in the di-IDP complex. The latter prediction was confirmed by re-examining the published crystal structure. IDP binding to one subunit enhanced the structural asymmetry, surprisingly, by changing the vacant subunit structure. The most significant asymmetrical change caused by IDP binding was a ‘rigid body’-like displacement of the lumenal loop connecting α-helices 2 and 3. This loop is found and highly conserved only in plant vacuolar mPPases and may have a regulatory function, such as pH sensing in the vacuole. In this regard, it will be of interest to determine the mPPase structure under acidic conditions.

Our findings further support the notion that the energy released on substrate binding in subunit A is stored as a conformational strain in subunit B. The stored energy may facilitate PP_i_ hydrolysis, product release, or H^+^ transport in subunit A. According to this concept, the enzyme dimer performs asymmetrically—one subunit carries out PP_i_ hydrolysis and ion transport while the other acts as a transient energy store. As our data define the conformational changes associated with substrate binding only, it will be interesting to determine in future studies how each mPPase subunit performs in the hydrolysis, product release, and transport steps. 

## Data Availability

The data presented in this study are available on request from the authors.

## Supplementary Materials

All data are available online at https://www.mdpi.com/article/10.3390/ijms222312902/s1.
