# Supplementary material for: A Lumenal Loop Associated with Catalytic Asymmetry in Plant Vacuolar H+-Translocating Pyrophosphatase
Source: Int J Mol Sci. 2021 Nov 29;22(23):12902. doi: 10.3390/ijms222312902 (PMC8657866; doi:10.3390/ijms222312902)
Supplement: Supplementary file 1 [file ijms-22-12902-s001.zip › ijms-1469213-supplementary.pdf]

Supplementary Material for:

"A lumenal loop associated with catalytic asymmetry in plant vacuolar H^+-translocating pyrophosphatase"

Viktor A. Anashkin and Alexander A. Baykov

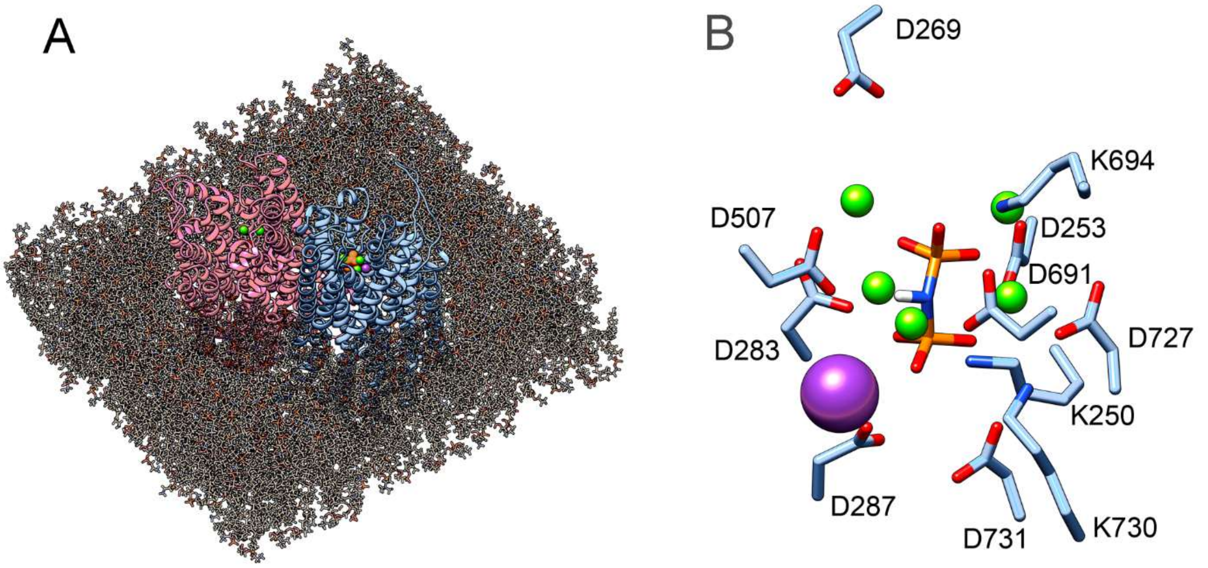

Figure S1. The modeled structure of Vr-mPPase dimer with IDP in one subunit. *A*, Dimeric enzyme in a lipid bilayer membrane. Ligands are shown as spheres: IDP is orange, Mg^{2+} is green, and K^+ is magenta. *B*, Active site with bound ligands shown in the same colors.

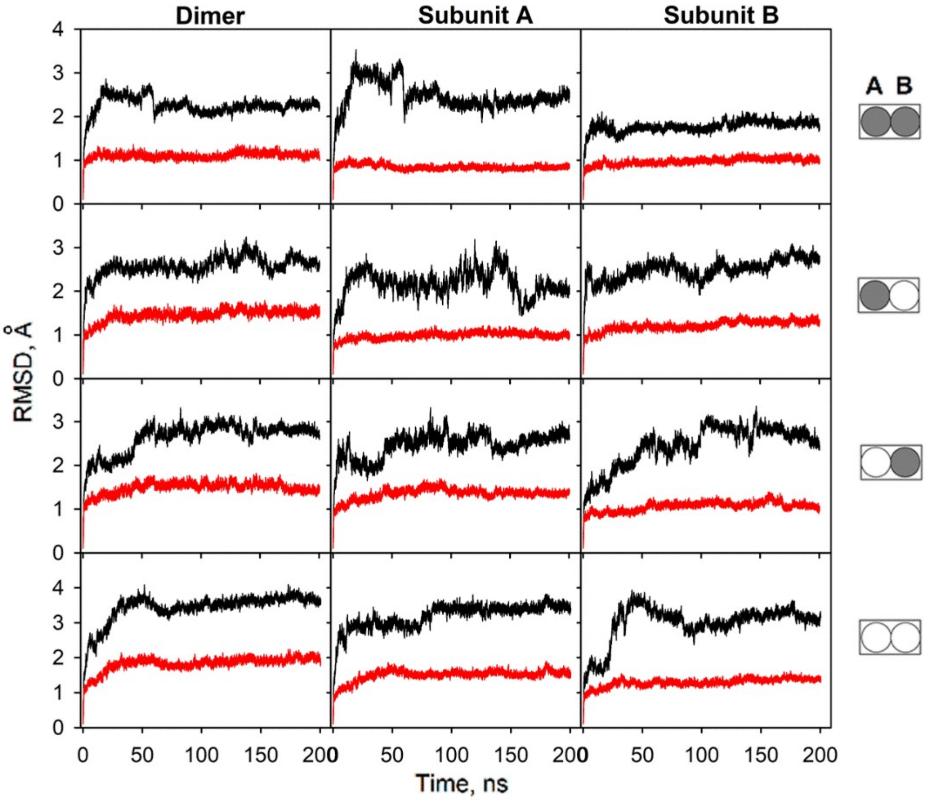

Figure S2. RMSD changes during MD simulations. RMSD values versus the crystal structure are for the backbone atoms in the entire dimers (*black curve*) and the transmembrane helices

only (*red curve*). The schemes on the right indicate the binding state of the subunits within the dimer (IDP-bound subunits are shown as closed circles).

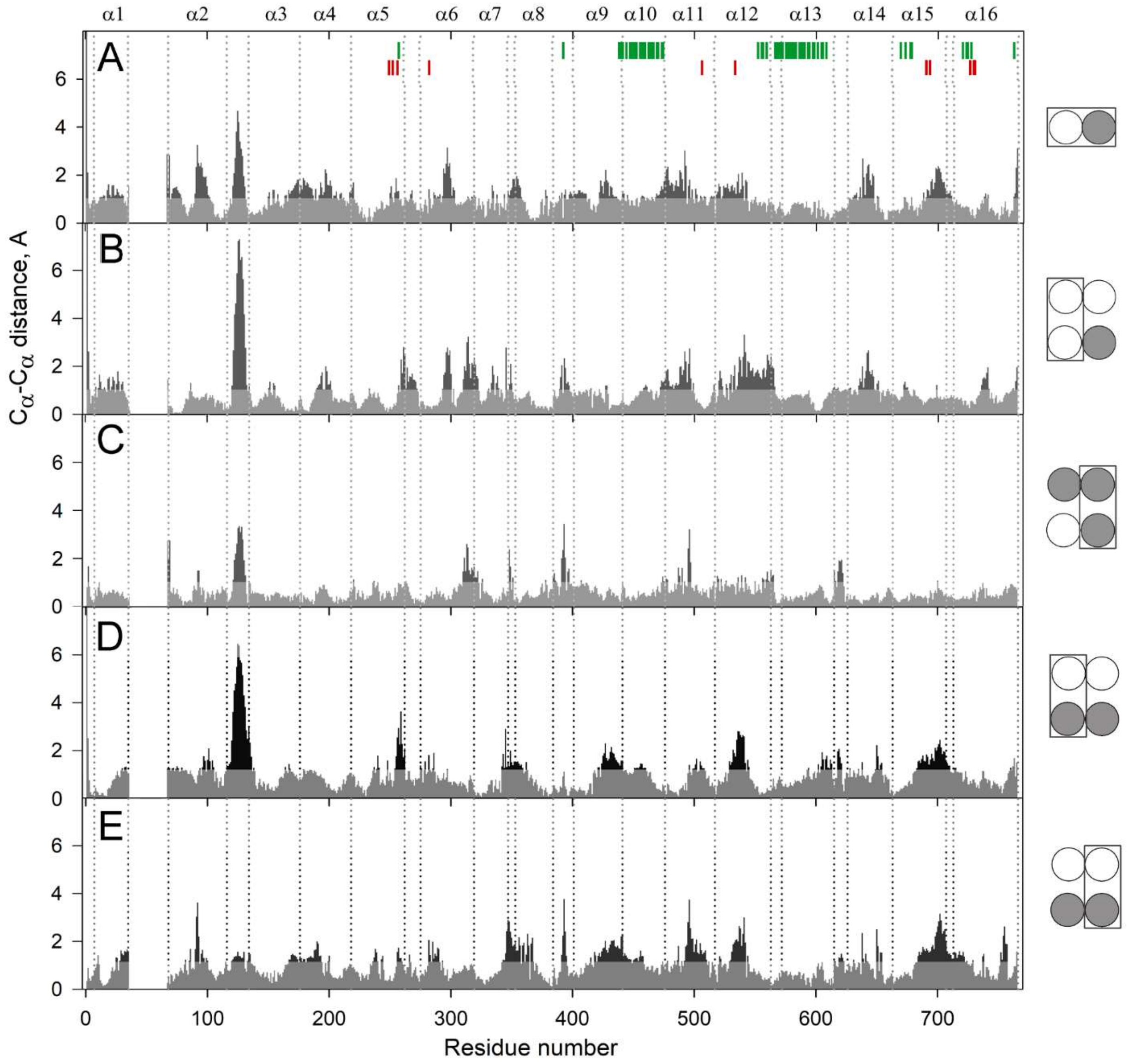

Figure S3. C_α atom displacements between superimposed MD-simulated subunit structures of the IDP complexes of Vr-mPPase of different stoichiometry. Three top panels differ from the three bottom panels in Fig 7 in that subunits A and B were interchanged. The structures compared were averaged from each 30th of the 4,000 snapshoots obtained between 160 and 200 ns of the MD simulations. The schemes on the right indicate the binding state of the subunits that were compared within dimer (three top panels) or between dimers (two bottom panels). IDP-free and IDP-bound subunits are shown as open and closed circles, respectively, the superimposed subunits are boxed. Vertical dotted lines separate the residues belonging to different α-helices, as indicated above panel *A*. Green and red vertical bars on the top panel mark the residues belonging to the active site and subunit contact, respectively.

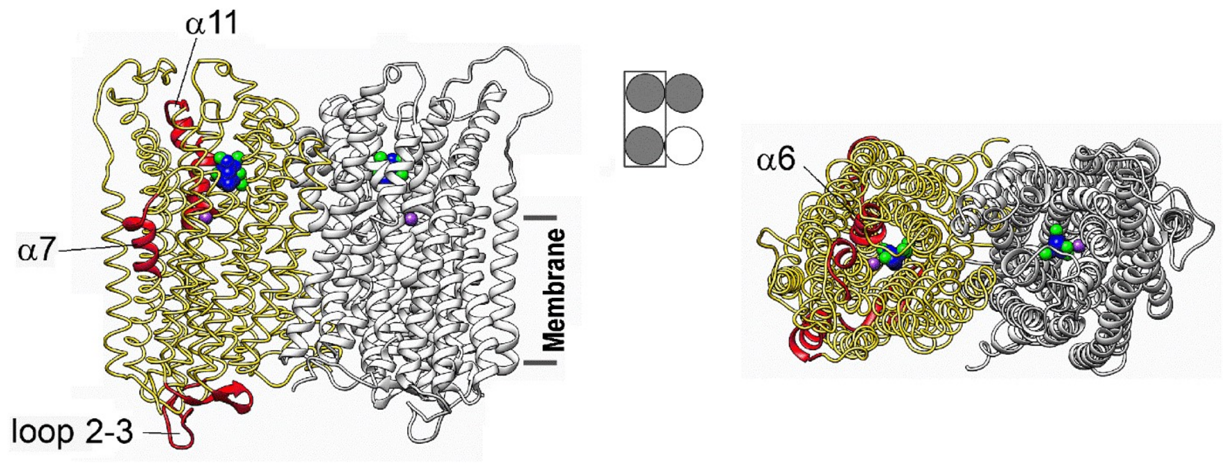

Figure S4. The regions undergoing the most significant displacements in the IDP-containing left subunit upon IDP binding to the right subunit. Red color marks the regions that will further change upon IDP binding to the right subunit. IDP molecule, five Mg^{2+} ions, and K^+ ion are depicted in a space-filling mode (blue, green, and magenta, respectively).

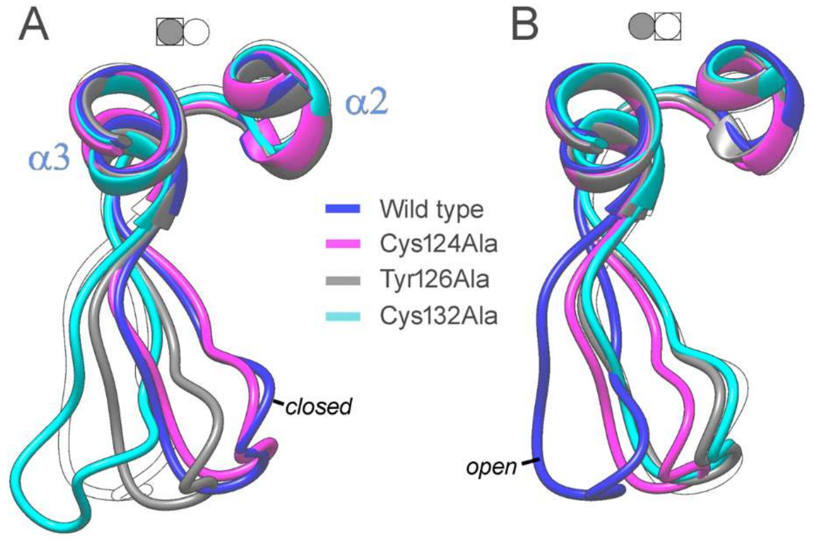

Figure S5. The conformations of loop 2–3 observed in the simulated structures of the mutant Vr-mPPases containing IDP in one subunit. *A*, The IDP-bound subunit. *B*, The IDP-free subunit. The uncolored chain in each panel shows the alternative loop 2–3 conformation occuring in wild-type Vr-mPPase. The schemes on the top define dimer occupancy with IDP (closed circle) and the subunit selected for loop 2–3 analysis (boxed circle).

Table S1. The intra-loop H-bonds supporting loop 2–3 structure in its two versions shown in Figure 6. The data refer to IDP-free subunits in the Vr-mPPase dimers with the indicated IDP content. The choice of the subunits compared is thus similar to that in Figure 2D. Bond occurrence is the percentage of the snapshots with the indicated H-bonds (donor-acceptor distance 2.8–3.1 Å) between 160 and 200 ns of the MD simulation.

Bond number In Figure 6	Donor–acceptor pair	Bond occurrence, %	
		No IDP bound	IDP in subunit A
1	Asp127(O) – Lys130(N)	87	95
2	Cys124(N) – Lys130(O)	87	90
3	Gln122(O) – Cys132(N)	90	96
4	Gln122(N) – Cys132(O)	87	84
5	Asp126(Oδ) – Thr129 (Oγ)	94	98

Table S2. The external H-bonds stabilizing loop 2–3 in its two positions shown in Figure 6. Bond occurrence is the percentage of the snapshots with the indicated H-bonds (average donor-acceptor distance 2.8–3.1 Å) between 160 and 200 ns of the MD simulation.

Subunit occupancy by IDP in dimer*	Subunit	Glu225(Oε)···Arg562(Nω1, Nω2, Nε)			X···Asp219(Oδ)			Asp218(O)···His319(Nε)	Loop 2–3 conformation
		Arg562 atom	Bond occurrence, %		Residue X (atom)	Bond occurrence, %		Bond occurrence**, %	
			With a particular atom	With the whole residue		With a particular atom	With the whole residue**		
		Nω1	**0**		Lys133(N)	33.8			
	A	Nω2	99.8	100	Lys133(Nε)	31.7	60.5	71.9	Closed
		Nε	97.6						
A ○○ B		Nω1	**0**		Lys133(N)	35.6			
	B	Nω2	100	100	Lys133(Nε)	42.8	65	74.6	Closed
		Nε	97.9						
		Nω1	**0**		Lys133(Nε)	53			
	A	Nω2	99.3	100	Lys133(N)	12.5	64	**11.8**	Closed
●○		Nε	96.8						
		Nω1	70.5		Lys133(N)	94			
	B	Nω2	**27.3**	89.3			94	**<1**	Open
		Nε	**14.8**						
		Nω1	**0**		Ser125(Oγ)	45.3	**45.5**		
	A	Nω2	80.3	88.8	Ser125 (N)	34.5		10.8	Open
		Nε	66.3		Lys133(N)	30.5	**30.5**		
●●		Nω1	0		Lys133(N)	56.3	64.8		
	B	Nω2	99.3	100	Lys133(Nε)	12.5		65.8	Closed
		Nε	98		Thr131(Oγ)	10.5	**10.5**		

* Open and closed circles refer to IDP-free and ID-bound subunits, A and B designate the left and right subunits, respectively.

** Bond occurrences less than 50 % (no stable bond exists) are shown in bold.

Table S3. Conservation of the intra-loop H-bonds in variant Vr-mPPases. The data refer to the Vr-mPPase dimers formed by IDP-bound subunit A and IDP-free subunit B. Bond occurrence is the percentage of the snapshots with the indicated H-bonds (donor-acceptor distance 2.8–3.1 Å) between 160 and 200 ns of the MD simulation.

Bond number in Figure 6	Donor–acceptor pair	Bond occurrence, %							
		Wild type		Cys124Ala		Tyr126Ala		Cys132Ala	
		A	B	A	B	A	B	A	B
1	Asp127(O)···Lys130(N)	93	95	91	93	87	89	71	94
2	Cys124(N)···Lys130(O)	96	90	89	88	89	93	**0**	94
3	Gln122(O)···Cys132(N)	99	96	87	79	98	100	54	83
4	Gln122(N)···Cys132(O)	80	84	74	76	88	90	76	83
5	Asp126(Oδ)···Thr129 (Oγ)	100	98	98	100	98	94	84	98

Table S4. The E263···R609··· R441' H-bond triad at the subunit interface is formed only in the IDP-containing subunit. Bond occurrence is the percentage of the snapshots with the indicated H-bonds (donor-acceptor distance <3.5 Å) between 160 and 200 ns of the MD simulation.

IDP-bound subunit	Analyzed subunit	Bond occurrence, %	
		Glu263(Oε)···Arg609(Nω)	Arg609(Nω)···Arg441′(O)*
None	A	<1	<1
	B	<1	<1
A	A	83	28
	B	<1	<1
B	A	39	14
	B	96	72
A and B	A	100	42
	B	99.5	78

*R441′ belongs to the other subunit.
